# Superficial and deep zone articular chondrocytes exhibit differences in actin polymerization status and actin-associated molecules *in vitro*

**DOI:** 10.1016/j.ocarto.2020.100071

**Published:** 2020-05-18

**Authors:** Elizabeth Delve, Vivian Co, Rita A. Kandel

**Affiliations:** a Lunenfeld-Tanenbaum Research Institute, Mount Sinai Hospital, Toronto, ON, Canada; b Institute of Biomaterials and Biomedical Engineering, University of Toronto, Toronto, ON, Canada; c University of Ontario Institute of Technology, Oshawa, ON, Canada; d Laboratory Medicine and Pathobiology, University of Toronto, Toronto, ON, Canada; e Pathology and Laboratory Medicine, Mount Sinai Hospital, Toronto, ON, Canada

**Keywords:** Superficial zone chondrocyte, Deep zone chondrocyte, Actin cytoskeleton, MRTF-A, YAP/TAZ

## Abstract

**Objective:**

The actin cytoskeleton regulates cell shape and plays a role in regulating chondrocyte phenotype. Most studies investigating regulation of the chondrocyte phenotype by the actin cytoskeleton use chondrocytes isolated from full-thickness (FT) cartilage, which has a heterogeneous cell population. Superficial zone chondrocytes (SZC) have an elongated morphology and account for 10–20% of chondrocytes, while the remaining chondrocytes in the deeper zones appear more rounded. This study characterizes the actin cytoskeleton and expression of actin-associated molecules in SZC and deep zone (DZ) chondrocytes (DZC) *in vitro* in order to identify molecules differentially expressed by SZC and DZC that may contribute to the observed differences in zonal chondrocyte shapes.

**Design:**

SZ, DZ, and FT chondrocytes isolated from bovine metacarpal-phalangeal joints were cultured in monolayer for 48 h*.* Macroscopic morphology, actin polymerization status, and expression of select actin-associated molecules (adseverin, cofilin, transgelin, vinculin, MRTF-A, and YAP/TAZ) were determined.

**Results:**

SZC appeared more elongated and have more filamentous actin compared to DZC, as determined by quantifying cell circularity and G-/F-actin ratio. MRTF-A gene and protein levels were significantly higher in SZC compared to DZC while DZC more highly expressed transgelin and TAZ. Although there was differential gene expression, no significant differences in adseverin, cofilin, vinculin, or YAP protein levels were observed between the two cell populations.

**Conclusions:**

This study identifies differences in actin polymerization status and expression of actin-associated molecules in primary SZC and DZC *in vitro*. These findings further our understanding of candidate actin-related pathways that may be regulating zonal chondrocyte phenotype.

## 1 Introduction

Articular cartilage has a stratified zonal architecture comprised of the superficial zone (SZ), the mid-zone (MZ), and the deep-zone (DZ), each with distinct matrix composition and organization [1]. Chondrocytes of the different zones exhibit unique cellular morphologies with SZ chondrocytes (SZC) appearing more elongated compared to the rounded cell morphology observed in the deeper zones [2], yet our understanding of the regulatory pathways governing the primary chondrocyte phenotype is derived largely from *in vitro* studies using chondrocytes isolated from full-thickness cartilage. These studies demonstrate that a round morphology and cortically arranged actin is required for the expression of the bulk matrix molecules collagen type II (col2) and aggrecan (agg) [[3], [4], [5]]; however, zone specific molecules are also present, such as proteoglycan 4 (PRG4) and clusterin (CLU) in the SZ [6,7]; and collagen type X (ColX) and alkaline phosphatase (ALP) in the DZ [8,9]. Little is known about the differential expression of molecules in SZC and DZ chondrocytes (DZC) that maintain differences in cell morphology and the zonal chondrocyte phenotype.

The polymerization status of the actin cytoskeleton has been shown to correlate with cell shape *in vitro* and has been identified as a critical regulator of both the primary full-thickness chondrocyte phenotype (col2 and agg) [3,10,11], as well as the primary SZ chondrocyte (SZC) phenotype (PRG4) [12,13]. One important role of the actin cytoskeleton is to allow the cell to transduce extracellular cues to intracellular signaling pathways by forming a physical link with the cell membrane via focal adhesion complexes [14,15]. Downstream, regulation of actin polymerization status is tightly controlled by actin-modifying proteins that affect actin dynamics by functioning to cap, sever, nucleate, and/or elongate actin filaments (F-actin) which are formed from actin monomers (G-actin) [16]. While hundreds of actin-modifying proteins have been identified, only a select few have been investigated in chondrocytes including adseverin (ads), cofilin (cfl), and transgelin (tagln). Ads and cfl are both members of the gelsolin superfamily of actin-modifying proteins, which not only function to cap and sever actin filaments but can also sequester actin monomers leading to actin depolymerization [17]. In primary full-thickness chondrocytes ads appears to play a role in depolymerizing the actin cytoskeleton as knock-down of ads increases levels of filamentous actin resulting in a decrease in the ratio of G-actin to F-actin [18]. While the role of cfl has not been investigated in primary chondrocytes, in passaged chondrocytes knock-down of cfl appears to promote actin polymerization resulting in a decreased G-/F-actin ratio [3]. Unlike ads, which decreases with cell passaging, cfl expression is elevated in passaged chondrocytes [3,18]. The effect of tagln, a member of the calponin family, on actin polymerization in chondrocytes has not yet been investigated but in other cell types tagln functions as an actin cross-linking protein [19]. Wilson et al. have reported the absence of tagln expression in mature chondrocytes [20]. Similarly, Parreno et al. observed the absence of tagln in primary full-thickness chondrocytes; however, expression increased with cell passaging [21]. Transgelin is also more highly expressed in chondroprogenitors compared to articular chondrocytes [22]. The differential expression of ads, cfl, and tagln has not yet been investigated in the zonal subpopulations of primary chondrocytes. While the pathways linking actin polymerization to gene transcription have only been partially elucidated in articular chondrocytes, one mechanism of action is via the cytoplasmic/nuclear shuttling of actin-regulated transcription factors myocardin-related transcription factor-A (MRTF-A), yes-associated protein (YAP), and transcriptional co-activator with PDZ-binding motif (TAZ) [13,21,23]. Cytoplasmic and nuclear localization of MRTF-A and YAP/TAZ are both regulated by actin polymerization status but the regulation of subcellular localization occurs through distinct mechanisms [24,25]. Full-thickness chondrocytes lack expression of MRTF-A but expression increases with cell passaging and has been shown to play a role in the acquisition of the dedifferentiated chondrocyte phenotype [10,21]. Conversely, YAP/TAZ is expressed by full-thickness chondrocytes *in vitro* and has been shown to be chondroprotective in an experimental osteoarthritis model in mice [21,26]. Both MRTF-A and YAP/TAZ are expressed by SZC and play a role in the maintenance of the SZ phenotype *in vitro* [13,23]. Little is known about the differential expression of these actin-regulated transcription factors in the zonal subpopulations.

The present study aims to characterize the polymerization status of the actin cytoskeleton and expression of select actin-associated molecules present in SZ and DZ subpopulations, which may contribute to our understanding of the molecular mechanisms regulating the distinct phenotype of primary zonal subpopulations; and to our understanding of how to recapitulate the phenotype of the zonal subpopulations *in vitro* for tissue engineering applications.

## 2 Methods

### 2.1 Cell culture

SZ, DZ, and FT chondrocytes were differentially isolated from bovine metacarpal-phalangeal articular cartilage aged 6–9 months by serial protease and collagenase enzymatic digestion as described previously [13]. Cartilage from the top 10–20% was isolated for the SZ and the lower 30–40% of the tissue for the DZ. Enrichment of the chondrocyte subpopulations was confirmed in freshly isolated cells by determining the differential gene expression of the zone specific markers PRG4 (SZ) and ColX (DZ) by qPCR; and the enriched ALP activity in the DZ compared to the other zones (data not shown). SZ, DZ, and FT chondrocytes were seeded individually in monolayer culture (density of 5.2 × 10^3^ cells/cm^2^) in high glucose DMEM supplemented with 1% ITS (354351; BD Biosciences; San Jose, CA, US), 100 nM dexamethasone, 40 μg/mL proline, 100 mM pyruvate, 100 μg/mL ascorbic acid, and maintained in a humidified incubator at 37 °C with 5% CO_2_ for 48 h prior to harvesting for analysis. Parreno et al. demonstrated that FT chondrocytes begin to dedifferentiate by day 3 in monolayer culture, which is why a 48 h time point was selected for these studies [27].

### 2.2 Quantification of cell area and circularity

After 48 h in monolayer culture, chondrocytes were incubated with calcein-AM (SC-203865 Santa Cruz Biotechnology; Dallas, Texas, USA) diluted in PBS−/− (without calcium or magnesium) (1:5000) for 10 min in the dark at room temperature. Images were acquired using a Leica fluorescent DM IL microscope and ImageJ analysis software was used to quantify cell area and circularity as described previously [13]. A minimum of 30 cells was analyzed from triplicate cultures per condition; and the experiment was repeated 4 times.

### 2.3 Quantification of cytoskeletal polymerization

Monomeric (G-actin and M-tubulin) and polymerized (F-actin and P-tubulin) fractions were isolated using the differential Triton solubility method as described previously [13]. Adherent chondrocytes seeded in a 6-well plate were incubated in monolayer culture with 100uL extraction buffer per well (1% Triton X-100 in PBS−/−) plus complete protease inhibitor (Roche Diagnostics; Manheim, Germany) to isolate the Triton soluble fraction containing predominantly the G-actin and M-tubulin portion. The solution was centrifuged and an equal volume of RIPA buffer (50 mM Tris HCl, 150 mM NaCl, 1% NP-40, 0.5% sodium deoxycholate, and 0.1% SDS) plus complete protease inhibitor (Roche Diagnostics; Manheim, Germany) was used to isolate the Triton insoluble fraction containing predominantly the F-actin and P-tubulin portion. Both monomeric and polymerized fractions were agitated for 30 min at 4 °C prior to the addition of 2× Laemmli buffer (4% SDS, 10% 2-mercaptoethanol, 20% glycerol, 0.004% bromophenol blue, and 0.125 M Tris-HCl). Samples were heated for 10 min at 98 °C and equal volumes were separated by SDS-PAGE. ImageJ analysis was used to perform densitometry on the resultant bands. Monomeric (G-actin and M-tubulin) and polymerized (F-actin and P-tubulin) fractions are presented as a percent of the total (sum of monomeric and polymerized fractions) for each condition.

### 2.4 RNA extraction and RT-PCR

RNA was extracted using TRIzol, as described by the manufacturer. Five hundred or 1000 ng of RNA was reverse transcribed with Superscript III (Life Technologies; Carlsbad, CA, USA). Gene specific primer sequences (listed in Table 1 were used to perform polymerase chain reaction (qPCR) with a LightCycler 96 Real-Time PCR system (Roche). The Pfaffl method was used to determine the mean relative quantification values, using 18S rRNA as the housekeeping gene [28].

**Table 1:** Gene-specific primer sequences.

Gene	Primer Sequence
18S rRNA	F: 5′-GTAACCCGTTGAACCCCATT-3′ R: 5′-CCATCCAATCGGTAGTAGCG-3′
Proteoglycan 4 (PRG4)	F: 5′-ATGCCTGAACCGACTCCTAC-3′ R: 5′-TGCCGAAGCCTTGACTGG-3′
Clusterin (CLU)	F: 5′-CGGTGACCGAGGGGT-3′ R: 5′-TTCCTGGAGCTCTTTGTCCG-3′
Collagen Type X (ColX)	F: 5′-ACAAGGACCTACAGGAGAAC-3′ R: 5′-GCGGCAAGGAGTACAATG-3′
Alkaline Phosphatase (ALP)	F: 5′-AAGCACTCTCACTATGTCTG-3′ R: 5′-GTCGCATTGTTCCTGTTG-3′
Vinculin (VCL)	F: 5′-CCCCAGAGGCCCGAGCATTA-3′ R: 5′-TGGCAGGTCTGCTGTTGGCT-3′
Adseverin (Ads)	F: 5′-ACCTCCGCATTCCTGACTG-3′ R: 5′-CAGACCTTCTTTCTTTGATGTTCC-3′
Transgelin (Tagln)	F: 5′-GAAGGTGCCCGAGAACCCGC-3′ R: 5′-ACCGCAGCCAGGTCTTTGCC-3′
Cofilin (Cfl)	F: 5′-CCCCTGAGTGTGCACCCCTTAA-3′ R: 5′-CCCCCAGCTTCTCTGCAAGGGT-3′
MRTF-A	F: 5′-GCCTCTACCTCACCATCC-3′ R: 5′-GCACTACACCAAGACACTAAG-3′
YAP	F: 5′-CTCTCCCCGAAACGCAGT-3′ R: 5′-AAGCAATTTCAGCGGACTGTA-3′
TAZ	F: 5′-ATGGCAAGACCCTAGGAAGG-3′ R: 5′-CAAGATTCGGCTGAGACACG-3′

### 2.5 Protein harvest and Western blot analysis

Total protein was harvested in RIPA buffer (50 mM Tris HCl, 150 mM NaCl, 1% NP-40, 0.5% sodium deoxycholate, and 0.1% SDS) plus complete protease inhibitor (Roche Diagnostics; Manheim, Germany). Protein was quantified using the bicinchoninic acid protein assay (Thermo Scientific; Waltham, MA, USA), as described by the manufacturer. Equal amounts of protein were separated by SDS-PAGE and wet-transferred to PVDF membranes at 80V for 1.5hrs. Membranes were incubated with 5% skim milk (in PPS−/−) for a minimum of 30 min prior to overnight incubation with the primary antibody (Table 2; diluted in 5% skim milk). Following three washes in 0.005% Tween/PBS−/−, membranes were incubated for 1 h at room temperature with the HRP-conjugated secondary antibody diluted in 5% skim milk (1:10,000; Abcam; anti-mouse: ab97040 or anti-rabbit: ab6721). ECL Prime Western Blot Detection Reagent (GE Healthcare, Buckinghamshire, UK) was used to produce chemiluminescent signals detected using DiaFilm Autoradiography Film (Diamed, Mississauga, CA); and these were semi-quantified using ImageJ analysis software.

**Table 2:** List of primary antibodies for Western blots.

Protein	Dilution	Manufacturer	Clonality
Actin	1/1000	Millipore (MAB1501)	monoclonal
Tubulin	1/1000	Sigma (T5168)	monoclonal
Adseverin	1/1000	Abcam (ab96105)	polyclonal
Transgelin	1/1000	GeneTex (GTX113561)	polyclonal
Cofilin	1/1000	Abcam (ab42824)	polyclonal
Vinculin	1/1000	Sigma (hVIN-1)	monoclonal
MRTF-A	1/1000	Santa Cruz (sc-21558)	polyclonal
YAP/TAZ	1/500	Cell Signalling (8418S)	monoclonal
GAPDH	1/3000	Millipore (ABS16)	polyclonal

### 2.6 Immunocytochemistry

Chondrocytes cultured on glass slides were washed once with PBS−/− and fixed in 4% paraformaldehyde (in PBS−/−) at room temperature for 10 min. Cells were permeabilized with 10% goat serum diluted with 0.2% Triton/PBS−/− for 30 min. Actin staining was performed by incubating samples with DNase I (1:500; Molecular Probes; D12371) to visualize globular actin and Phalloidin-568 (1:20; Invitrogen; A12380) to visualize filamentous actin, in 3% goat serum diluted with 0.2% Triton/PBS−/− for 1 h at room temperature. Vinculin (VCL) staining was performed by incubating samples overnight with the primary antibody (1: 300; Sigma; hVIN-1) diluted in 3% goat serum diluted with 0.2% Triton/PBS−/−. Samples were washed three times and subsequently incubated with the secondary antibody anti-rabbit IgG conjugated Alexa Fluor 488 (1:250; Invitrogen; A11008) and Alexa Fluor 568 phalloidin conjugate (1:20; Invitrogen; A12380) to visualize filamentous actin, in 3% goat serum diluted with 0.2% Triton/PBS−/− for 1 h at room temperature. Samples were counterstained with 4′,6-diamidino-2-phenylindole (1:1500 in PBS−/−) for 5 min. Cells were washed and mounted using Permafluor Mountant (Thermo Scientific; Runcon, Cheshire, UK). Confocal microscopy was performed using a NikonC1si laser scanning confocal microscope.

### 2.7 Statistical analysis

Independent experiments were repeated 3–5 times using chondrocytes isolated from different batches of animals. Cells from multiple animals were pooled to obtain sufficient numbers to perform each experiment (N). Gene expression (performed in triplicate) and densitometry data is expressed as mean ± 95% confidence intervals. Cell area and circularity data is presented as box and whisker plots representing the minimum, mean, maximum, 25th and 75th percentiles. Grubb's test was used to identify outliers in the data sets using GraphPad QuickCalcs. Prism statistical analysis software was used to perform a one-way analysis of variance (ANOVA) followed by Tukey's Post Hoc test. Statistical significance was assigned at p < 0.05.

## 3 Results

### 3.1 Chondrocytes from the SZ, DZ, and FT exhibit different cell morphologies and actin organization *in vitro*

**Fig. 1:**
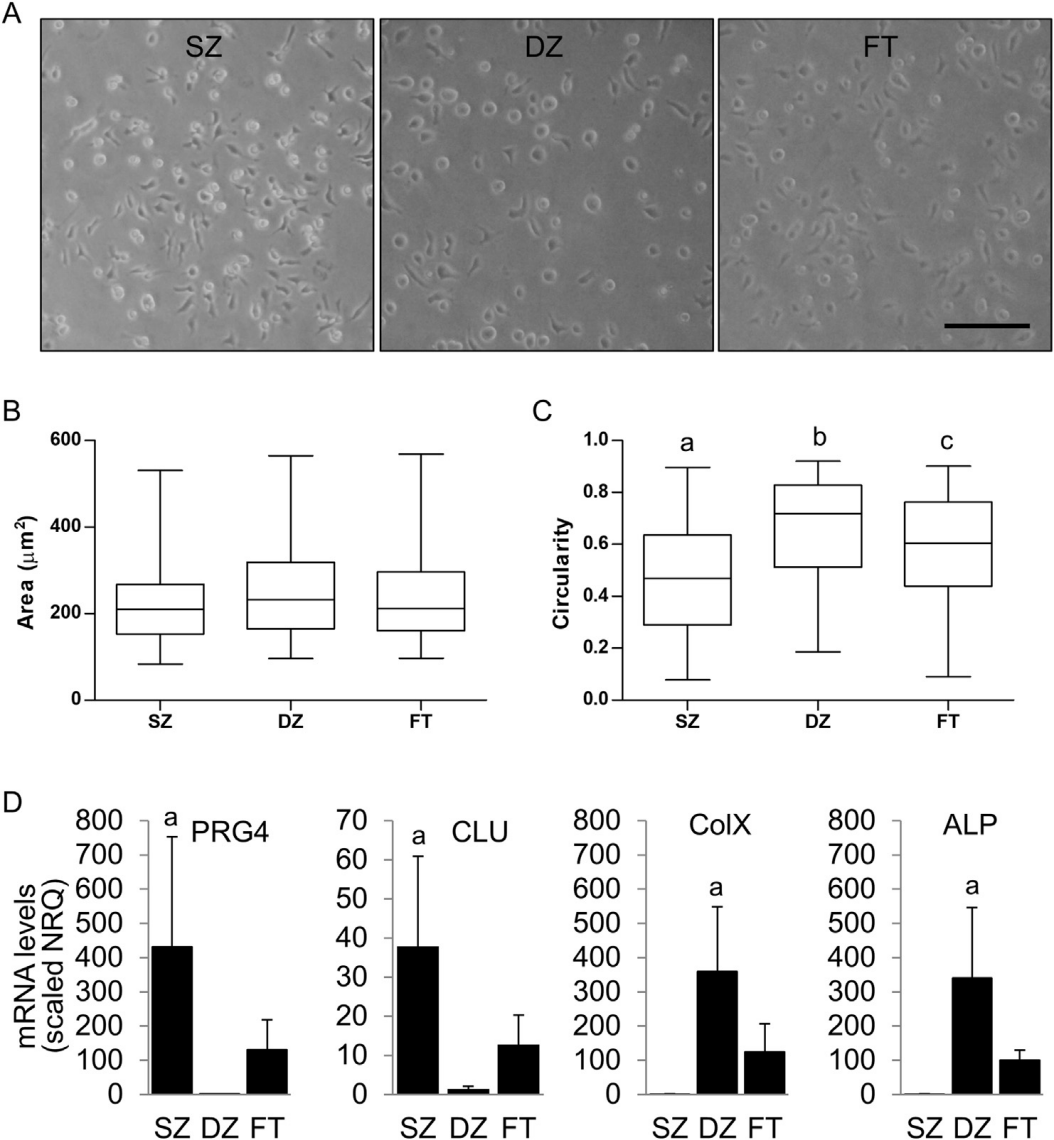
**Assessment of Morphology and Zonal Chondrocyte Phenotype in Monolayer Culture.** (A) Visualization of cells by phase contrast microscopy. Scale bar = 100 μm. Quantification of (B) cell area and (C) cell circularity. (D) mRNA levels of superficial zone (SZ) markers PRG4 and CLU and the deep zone (DZ) markers ColX and ALP. Analyses were performed at 48 h. N = 3. a, b, c = p < 0.05 and indicates significantly different from other groups.

SZC and DZC have different morphologies *in vivo* therefore we examined cell shape at 48 h to determine whether these cells maintained differences *in vitro.* Chondrocyte subpopulations exhibited different gross morphologies observed by phase contrast microscopy. SZ and FT subpopulations appeared to have more elongated cells compared to the DZ (Fig. 1A). Visual differences in appearances were confirmed by quantifying cell area and circularity. There were no significant differences in cell area between the subpopulations or compared to FT chondrocytes (Fig. 1B). However, significant differences in circularity were observed. SZ, DZ, and FT populations were all significantly different with the SZ subpopulation being the most elongated followed by FT, and DZ appearing the most rounded (SZ:DZ, DZ:FT, and SZ:FT; p < 0.0001) (Fig. 1C). To ensure chondrocyte subpopulations maintained their respective phenotypes in monolayer culture, the expression of zone-specific markers was assessed by qPCR. After 48 h in monolayer culture, the SZ subpopulation maintained elevated expression of PRG4 (SZ: DZ; p = 0.004; SZ:FT; p = 0.046) and CLU (SZ: DZ; p = 0.0007; SZ:FT; p = 0.017), and the DZ subpopulation maintained expression of ColX (DZ: SZ; p = 0.0002; DZ:FT; p = 0.01) and ALP (DZ: SZ; p = 0.0009; DZ:FT; p = 0.016) (Fig. 1D).

**Fig. 2:**
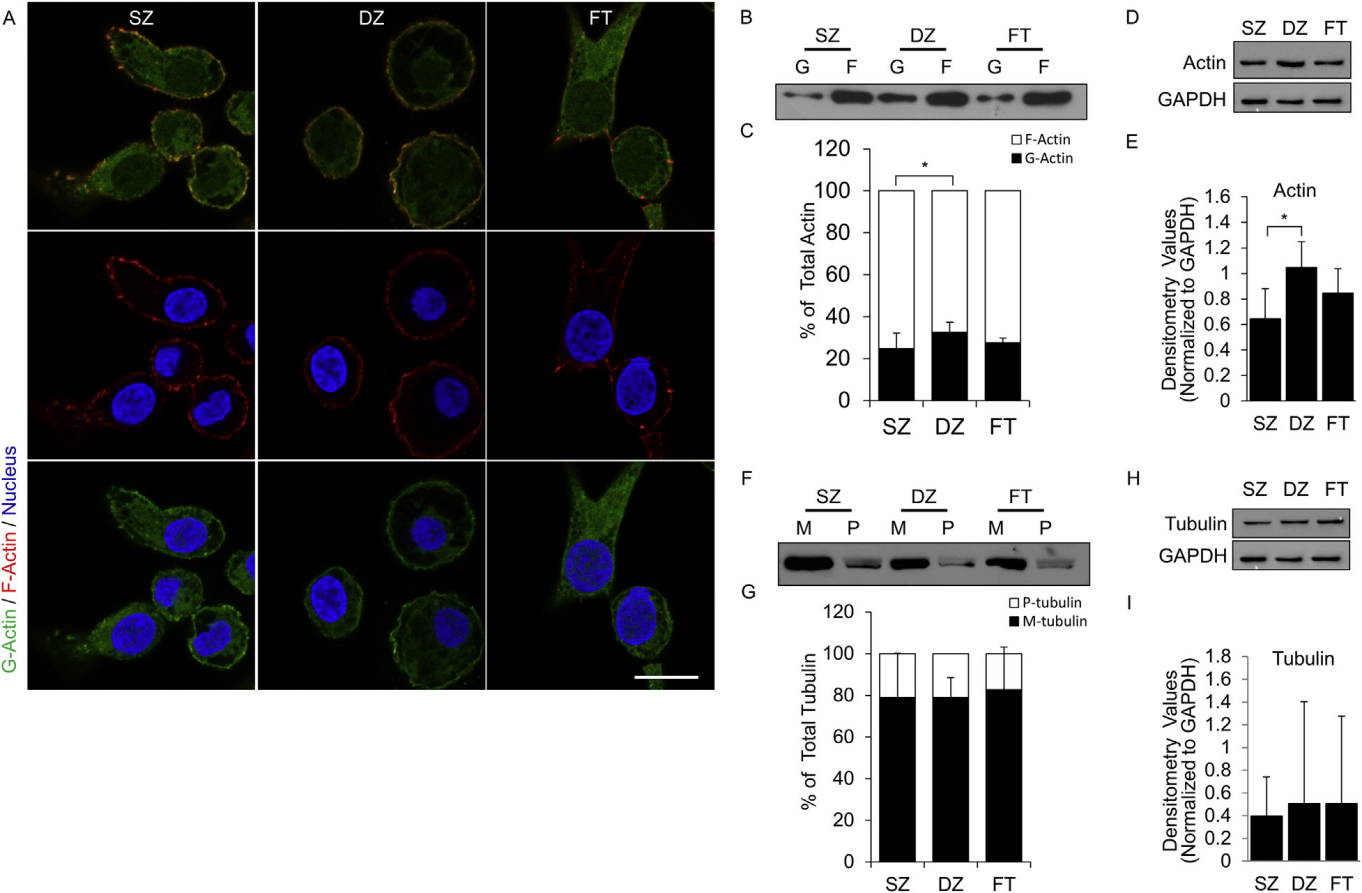
**Cytoskeletal Organization of Superficial Zone, Deep Zone, and Full-Thickness Chondrocytes.** Globular and filamentous actin assessed by (A) confocal microscopy. Green = Globular Actin. Red = Filamentous Actin. Blue = Nucleus. Scale bar = 10 μm. Actin polymerization status was assessed by determining the G-/F-actin ratio by (B) Western blot and (C) densitometry analysis. Total actin levels were determined by (D) Western blot and (E) densitometry analysis. Tubulin polymerization status was assessed by determining the M-/P-tubulin ratio by (F) Western blot and (G) densitometry analysis. Total tubulin levels were determined by (H) Western blot and (I) densitometry analysis. Analyses were performed at 48 h. N = 3–4. ∗ = p < 0.05 and indicates significance between groups.

As the cells maintained differences in morphology and expression of zone-specific markers at 48hrs of culture, the actin cytoskeleton status was examined. Confocal imaging of G- and F-actin showed the presence of both globular and filamentous actin in chondrocyte subpopulation; no obvious visual differences in actin distribution were observed. Quantification of the G-/F-actin revealed that the DZ subpopulation had a higher G-/F-actin ratio (p = 0.02) (Fig. 2B and C) and higher levels of total actin (p = 0.005) (Fig. 2D and E), compared to SZ. No significant differences in the G-/F-actin ratio or total actin were observed in FT population, as compared to SZ (p = 0.49) or DZ (p = 0.13) subpopulation. In addition to actin, microtubules are also part of the cellular cytoskeleton and can modulate cell shape and phenotype [29]. Thus, we investigated the polymerization status and the expression levels of tubulin microtubules between the chondrocyte subpopulations. We observed no differences in the ratio of monomeric tubulin (M) to polymerized tubulin (P) (Fig. 2F and G) and no differences in total tubulin levels between SZ, DZ, and FT subpopulations (Fig. 2H and I).

### 3.2 Zonal chondrocytes differentially express actin-associated molecules

**Fig. 3:**
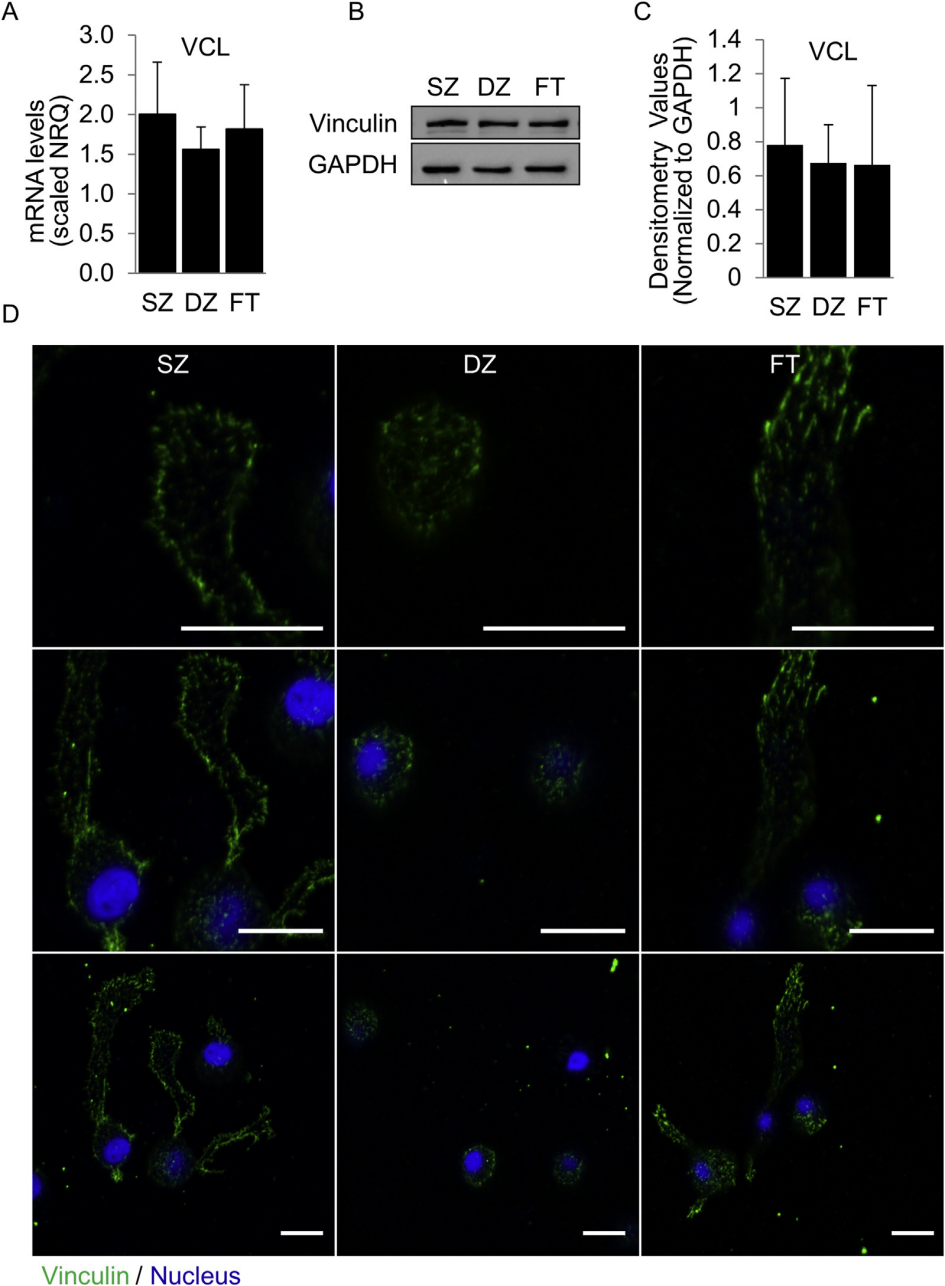
**Expression and Localization of Focal Adhesion Molecule Vinculin.** (A) mRNA levels of vinculin (VCL) in superficial zone (SZ), deep zone (DZ) and full-thickness (FT) chondrocytes. Total protein levels were determined by (B) Western blot and (C) densitometry analysis. (D) Visualization of vinculin distribution by confocal microscopy. Green = Vinculin. Blue = Nucleus. Scale bar = 10 μm. Analyses were performed at 48 h. N = 3. No significant differences were observed in expression levels. (For interpretation of the references to color in this figure legend, the reader is referred to the Web version of this article.)

Given the differences observed in actin polymerization status in SZ and DZ subpopulation, we evaluated the expression of actin-associated molecules that can influence actin polymerization. The accessory protein VCL, required for the formation of focal adhesions, allows the cell to sense the external environment and regulates actin polymerization by nucleating actin to promote the formation of actin filaments [30]. VCL mRNA levels and total protein levels did not vary significantly between subpopulations (Fig. 3); however, VCL localization did. In the SZ population VCL staining appears to indicate the presence of both focal contacts and more developed focal adhesions, while focal contacts appeared to predominate in the DZ subpopulation (Fig. 3). Cells that resembled the SZ and DZ subpopulations were both observed in the FT population.

**Fig. 4:**
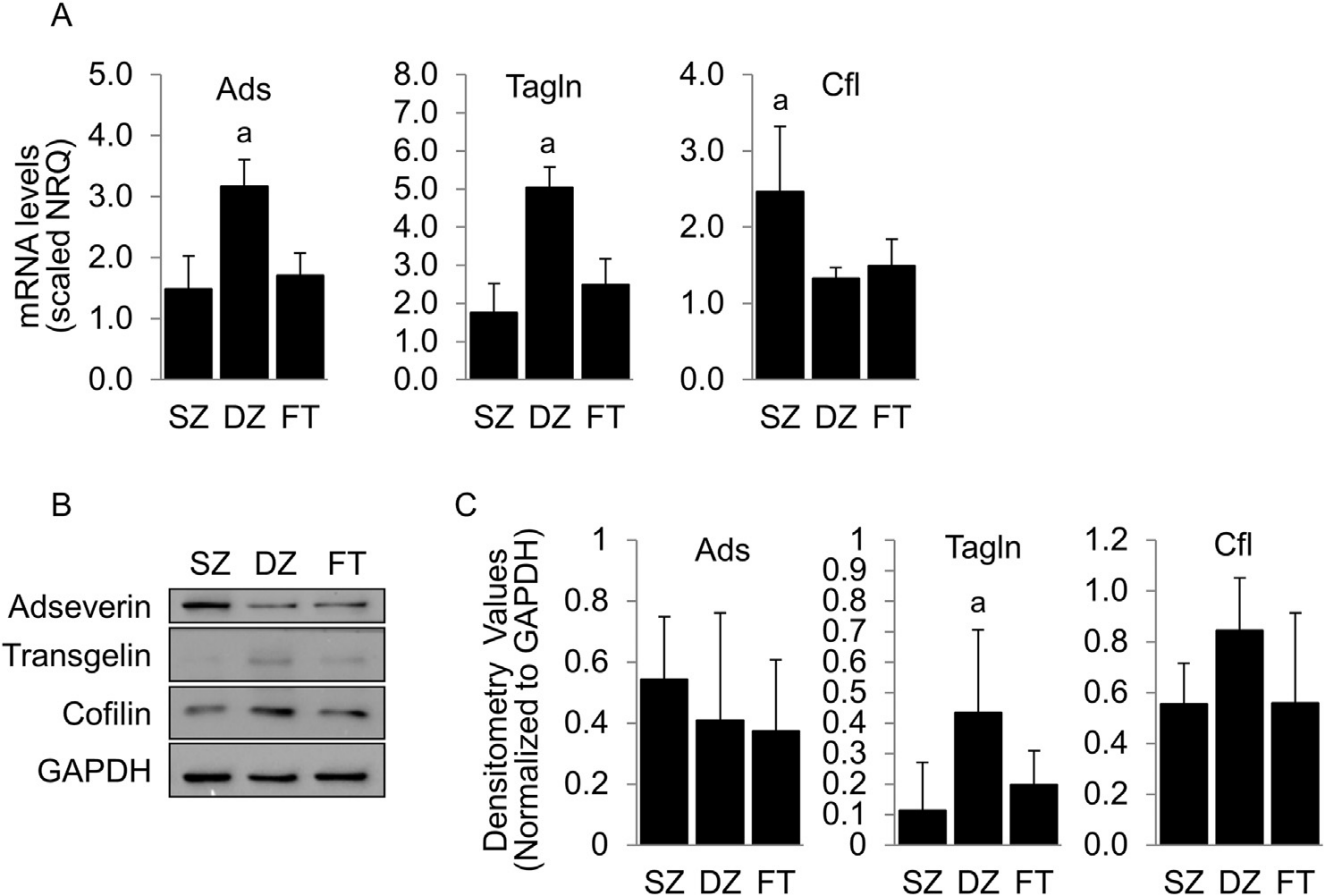
**Expression of Actin-Modifying Proteins Adseverin, Transgelin, and Cofilin.** (A) mRNA levels of adseverin (ads), transgelin (tagln) and cofilin (cfl) in superficial zone (SZ), deep zone (DZ), and full-thickness (FT) chondrocytes. Total protein levels were determined by (B) Western blot and (C) densitometry analysis. Analyses were performed at 48 h. N = 3–5. a = p < 0.05 and indicates significantly different from other groups.

Actin polymerization status can also be influenced by the expression of actin modifying proteins. We evaluated the expression of three actin-modifying proteins, adseverin (ads), cofilin (cfl), and transgelin (tagln), in chondrocyte subpopulations that are differentially expressed between primary (round) and passaged (elongated) chondrocytes isolated from full-thickness cartilage [3,18,21]. Ads mRNA levels were significantly higher in the DZ subpopulation compared to SZ (p < 0.0001) and FT (p = 0.0001) cells. However, no significant differences in ads protein expression was observed (Fig. 4). Tagln mRNA and protein expression were significantly elevated in the DZ subpopulation, compared to SZ (p < 0.0001) and FT (p < 0.0001) (Fig. 3). No significant differences in cfl protein levels were observed despite significantly higher mRNA levels in the SZ (SZ:DZ; p = 0.006; SZ:FT; p = 0.019) (Fig. 4).

### 3.3 Chondrocyte subpopulations differentially express MRTF-A and YAP/TAZ

**Fig. 5:**
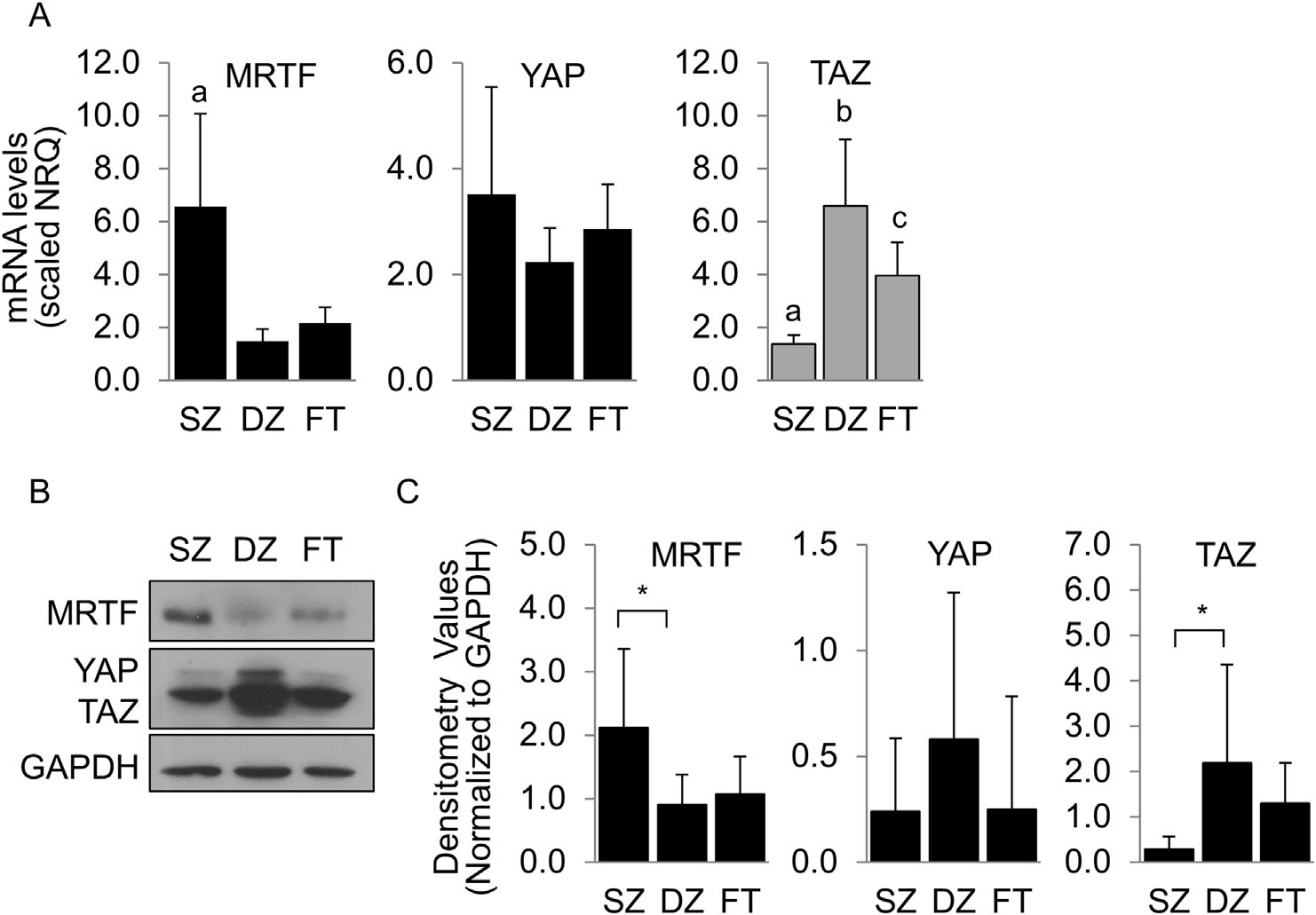
**Expression of Actin-Regulated Transcription Factors MRTF-A and YAP/TAZ.** (A) mRNA levels of MRTF-A, YAP, and TAZ in superficial zone (SZ), deep zone (DZ), and full-thickness (FT) chondrocytes. Total protein levels were determined by (B) Western blot and (C) densitometry analysis. Analyses were performed at 48 h. N = 3–4. a, b, c = p < 0.05 and indicates significantly different from other groups. ∗ = p < 0.05 and indicates significance between groups.

Actin polymerization status has been shown to effect downstream signaling pathways through actin-regulated transcription factors [31], therefore the levels MRTF-A and YAP/TAZ were determined in SZ, DZ, and FT subpopulations. SZ chondrocytes more highly express MRTF-A mRNA levels (p = 0.002) and protein levels (p = 0.03) (Fig. 5), compared to DZ. There were no significant differences observed in mRNA levels or protein levels for YAP in the two zones; however TAZ expression was significantly increased in the DZ subpopulation as compared to SZ mRNA levels (p < 0.0001) and protein levels (p = 0.013). Significant differences between FT and SZ (p = 0.049) and FT and DZ (p = 0.045) TAZ expression were only observed in mRNA levels but not protein levels (Fig. 5).

## 4 Discussion

This study demonstrated that primary SZ and DZ chondrocytes maintain distinct phenotypes *in vitro* at 48 h by exhibiting differences in actin polymerization status and expression of actin-associated molecules. The SZ subpopulation demonstrated a lower G-/F-actin ratio indicative of more polymerized actin and as well more highly expressed MRTF-A. Additionally, the DZ subpopulation more highly expressed the actin-modifying protein tagln and actin-regulated transcription factor TAZ. Although it is possible that these findings are not representative of chondrocytes *in vivo* the cells appeared to maintain their differentiation state as shown by two findings. Firstly, the cells maintained differences in cell shape *in vitro* as evidenced by the SZ subpopulation exhibiting a more elongated phenotype (less circular) than DZ cells. Secondly, the differential expression of zonal markers PRG4 and CLU in the SZ subpopulation and ColX and ALP in the DZ subpopulation was maintained at 48 h.

The finding of differential actin polymerization status in SZ compared to DZ subpopulations is not unexpected as SZ chondrocytes in native articular cartilage are more elongated compared to the rounded chondrocytes in the DZ [2]. Although Benya et al. showed that the actin cytoskeleton status (filamentous versus globular actin) can effect changes in chondrocyte phenotype in the absence of cell shape changes in passaged chondrocytes, we observed differences in both cell circularity and actin polymerization status in SZ and DZ subpopulations. While there were no differences in cell area measurements between subpopulations, the DZ subpopulation had higher levels of total actin suggesting DZ chondrocytes may have a larger cell volume, a parameter not examined in this study. Although FT circularity was significantly different from SZ and DZ, significant differences in the G-/F-actin ratio were not observed which is likely a reflection of the percentages of the zonal subpopulations in the FT cells.

Actin polymerization status has been shown to be an important regulator of the dedifferentiated chondrocyte phenotype acting in part via the actin-regulated transcription factor MRTF-A [10,21], while both MRTF-A and YAP/TAZ have been shown to regulate the SZ phenotype [13,23]. In this study differential expression of these transcription factors between the zonal subpopulations were observed. MRTF-A gene and protein were most predominantly detected in the SZ subpopulation with limited amounts in the DZ subpopulation. YAP/TAZ protein was detected in both SZ and DZ subpopulations; however, the DZC had significantly higher levels of TAZ mRNA and protein compared to SZ. Although a role for YAP/TAZ in the regulation of SZ phenotype has been demonstrated [23], the presence of TAZ in the DZ may in part play a role in regulating mineralization, a characteristic of DZC, which has been shown for other mineralizing cells [32,33]. Zhu et al. demonstrate TAZ up-regulation during osteogenic differentiation of adipose tissue derived stem cells in addition to impaired osteogenic differentiation and reduced ALP activity with TAZ knockdown [32]. YAP/TAZ has also been shown to play a role in the mineralization of enamel in mandibular molars in rats [33]. It is important to note that YAP/TAZ activity can be regulated by both canonical Hippo signaling and actin polymerization status [34,35]. While actin polymerization status has been shown to regulate YAP/TAZ localization in SZ chondrocytes [23], it is possible that the canonical Hippo cascade may also contribute to regulation of the primary zonal chondrocyte phenotype.

Although not significant, the actin-modifying protein ads had elevated protein levels in the SZ subpopulation. This was unexpected for two reasons. Firstly, over-expression of ads in non-hypertrophic epiphyseal plate chondrocytes promotes a polygonal morphology, increases cell volume, and up-regulates the expression of hypertrophic markers Indian hedgehog (Ihh) and ColX [36]. Secondly, adseverin knockdown in primary chondrocytes isolated from FT cartilage results in the formation of actin stress fibres, an elongated cell shape, and a decreased G-/F-actin ratio [18]. However, ads has been shown to facilitate the secretion of molecules in chromaffin cells, and may fulfil a similar function in SZ chondrocytes [37]. SZ chondrocytes express and secrete the zone-specific glycoprotein PRG4, which plays a critical role in maintaining articular cartilage homeostasis and limiting inflammation [38]. It is possible that ads modulates PRG4 secretion; however, the function of ads has not yet been investigated in primary zonal articular chondrocyte subpopulations requiring further study to elucidate its role. Interestingly, the elevated ads mRNA levels in the DZ subpopulation did not result in increased amount of protein, which may be a result of limited translation or enhanced degradation of the mRNA.

The increased tagln expression in the DZ subpopulation, compared to SZ was also unexpected. Firstly, tagln promotes actin polymerization as shown in vascular smooth muscle cells, which is a feature of SZ chondrocytes [39]. Secondly, knock-down of tagln in vascular smooth muscle cells promotes a spherical morphology and enhances chondrogenesis indicated by increased expression of col2, agg, and sex-determining region Y-box 9 (sox9) [39]. Conversely in leucocytes, tagln has been shown to stabilize the cortical actin ring [40], suggesting that tagln has tissue specific functions. Interestingly, although tagln negatively regulates the expression of the chondrogenic genes in vascular smooth muscle cells, Parreno et al. did not observe changes in col2 and agg expression with tagln knockdown in passaged chondrocytes [21]. The ratio between tagln and cfl may also be an important determinant in polymerization status and cell function as tagln can compete with cfl for F-actin binding [40]. Despite significantly higher levels of cfl mRNA in the SZ subpopulation, no significant differences were observed in cfl protein expression, despite elevated levels in DZ cells. Although the role of cfl in the zonal chondrocyte phenotype is not known, knockdown of cfl in passaged chondrocytes results in increased actin polymerization [3], suggesting cfl plays a role in depolymerization in passaged chondrocytes and raises the possibility that cfl may also play a role in actin depolymerization in DZ chondrocytes. Additionally, total protein levels alone may not indicate the activity of tagln and cfl, as phosphorylation of both molecules has been shown to influence their activity and actin regulation [41,42].

Cell shape is a differentiating feature between SZ chondrocytes and chondrocytes of the deeper zones. Although the actin cytoskeleton regulates cell shape and has been identified as a critical regulator of the primary chondrocyte phenotype, tubulin - another cytoskeletal element, also regulates cell shape [29]. Our lab previously demonstrated the polymerization status of tubulin microtubules did not contribute to the development of the passaged chondrocyte phenotype [3]; however, tubulin polymerization status has been shown to play a role in the primary chondrocyte phenotype by regulating sox9 expression and glycosaminoglycan production [43,44]. Although no differences in total tubulin levels or polymerization status were observed, this does not exclude a functional role in regulating the zonal chondrocyte phenotype.

Many studies elucidating the regulation of the primary chondrocyte phenotype use chondrocytes isolated from full-thickness cartilage. However, this study demonstrates that SZ and DZ chondrocytes exhibit unique differences in actin cytoskeleton, select actin related molecules, and actin regulated transcription factors that may contribute to the maintenance of the distinct zonal chondrocyte phenotype. Although a rounded morphology and depolymerized actin cytoskeleton favours the ‘primary chondrocyte phenotype’ in terms of col2 and agg expression, a unique approach may be required to recapitulate the distinct zonal chondrocyte phenotypes *in vitro* for tissue engineering and cell-based therapy applications.

## 5 Contributions

Conception and design: ED, VC, and RK. Analysis and interpretation of the data: ED, VC, and RK. Drafting the article: ED and RK. Critical revision and final approval of the article: ED, VC, and RK. Obtaining funding: ED and RK. Collection and assembly of data: ED and VC.

## Role of funding source

This work was funded by 10.13039/501100000024Canadian Institutes of Health Research (CIHR) Grant #MOP12611. ED was supported by the QEII-GSST Mount Sinai Scholarship in Science and Technology, Barbara and Frank Milligan Graduate Fellowship, and the Samuel Lunenfeld Research Institute OSOTF Award.

## Declaration of Competing Interest

The authors declare that there is no conflict of interest.
